# Professor of Surgery in Harvard University

**Published:** 1849-06

**Authors:** 


### PROFESSOR OF SURGERY IN HARVARD UNIVERSITY.

Dr. Hayward has resigned the professorship of Surgery in Harvard University, and Dr. Henry J. Bigelow has been appointed his successor. Dr. Bigelow is said to be the youngest man ever called to teach surgery in New England, but he carries with him to his responsible office a reputation already high for genius, learning, and surgical skill. He follows men who have conferred luster upon the American profession, but his friends are confident of his ability to sustain himself in his elevated position.

